# Approaches and Limitations in the Investigation of Synaptic Transmission and Plasticity

**DOI:** 10.3389/fnsyn.2019.00020

**Published:** 2019-07-24

**Authors:** Stephen D. Glasgow, Ryan McPhedrain, Jeanne F. Madranges, Timothy E. Kennedy, Edward S. Ruthazer

**Affiliations:** Department of Neurology & Neurosurgery, Montreal Neurological Institute-Hospital, McGill University, Montreal, QC, Canada

**Keywords:** synapse, electrophysiology, analysis, mEPSCs, evoked potential, LTP (long term potentiation), spontaneous release

## Abstract

The numbers and strengths of synapses in the brain change throughout development, and even into adulthood, as synaptic inputs are added, eliminated, and refined in response to ongoing neural activity. A number of experimental techniques can assess these changes, including single-cell electrophysiological recording which offers measurements of synaptic inputs with high temporal resolution. Coupled with electrical stimulation, photoactivatable opsins, and caged compounds, to facilitate fine spatiotemporal control over release of neurotransmitters, electrophysiological recordings allow for precise dissection of presynaptic and postsynaptic mechanisms of action. Here, we discuss the strengths and pitfalls of various techniques commonly used to analyze synapses, including miniature excitatory/inhibitory (E/I) postsynaptic currents, evoked release, and optogenetic stimulation. Together, these techniques can provide multiple lines of convergent evidence to generate meaningful insight into the emergence of circuit connectivity and maturation. A full understanding of potential caveats and alternative explanations for findings is essential to avoid data misinterpretation.

## Introduction

A fundamental basis of information transfer in the nervous system is the release of neurotransmitter from the presynaptic terminal into the synaptic cleft to activate receptors on the postsynaptic neuron. These receptors can activate downstream signaling processes and initiate ionic flux through receptor pores. This in turn can alter the transmembrane electrical potential, which propagates to the cell body and axon hillock to evoke an action potential (AP) in the postsynaptic neuron. Temporal and spatial summation play pivotal roles in regulating neuronal output, through the filtering and integration of synaptic events with a range of amplitudes and durations (Magee and Johnston, 2005; Spruston, 2008). Indeed, coincident pre- and post-synaptic firing can lead to forms of synaptic strengthening of connections between two or more neurons, which can last for seconds, minutes, hours, days, and even a lifetime (Bliss and Gardner-Medwin, 1973; Abraham et al., 2002). Through dynamic recruitment of postsynaptic receptor complexes and modulation of presynaptic vesicular release, these changes contribute to the cellular basis of learning and memory in the brain.

Synaptic transmission and plasticity have been studied using various approaches, including molecular biology, behavior, electrophysiology, and imaging, however no single method is without drawbacks. Electrophysiology provides high temporal resolution, on the order of fractions of milliseconds, while enabling the pharmacological investigation of synaptic physiology. However, extracellular electrophysiological techniques, such as field potential recording and spike sorting, suffer from relatively poor spatial resolution. Whole-cell electrophysiology provides high temporal and spatial localization, but is typically configured for single or simultaneous double neuronal recordings (though some labs have successfully recorded from up to 12 neurons in parallel), limiting its utility for understanding complex network-level interactions (Miles and Poncer, 1996; Markram et al., 1997; Gal et al., 2019). In contrast, biochemical techniques such as immunohistochemistry, *in situ* hybridization and single cell transcriptomics provide high neuroanatomical specificity, and can allow for quantitative comparisons, but exhibit relatively low temporal acuity. More recently, the development of optogenetics, genetically-encoded calcium indicators (GECIs) and voltage probes have provided methods for cell-type specific cellular, and even synaptic, interrogation (Fenno et al., 2011; Lin and Schnitzer, 2016). In this review, we discuss common electrophysiological techniques as well as integration of novel optical methods used to investigate synaptic transmission and plasticity in the developing and mature brain. We further discuss the interpretation and caveats associated with the use of these methods to investigate synaptic properties.

### From Networks to Synapses: Insights Into Synaptic Transmission

**Figure 1:**
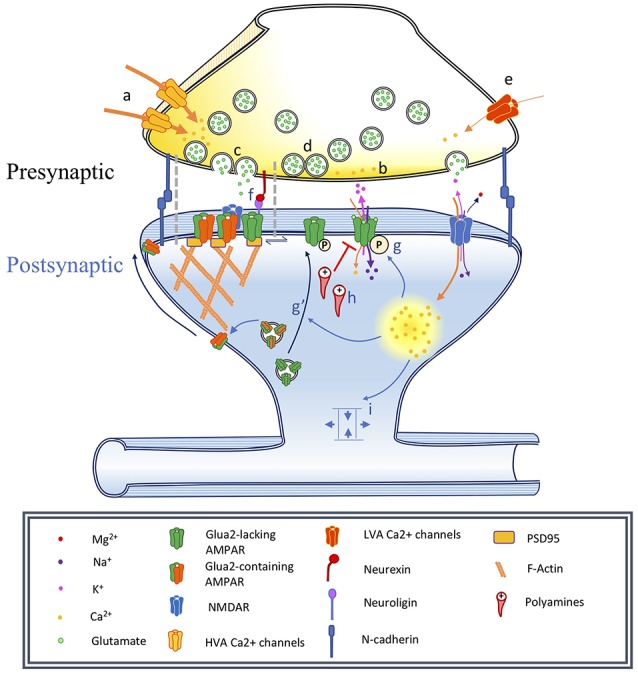
Changes associated with long-term potentiation and synaptic maturation. Under basal conditions synapses are adhesive junctions between a presynaptic terminal containing neurotransmitter vesicles and Ca^2+^-dependent release machinery, in close proximity to a postsynaptic site where various neurotransmitter receptors are present, often on electrotonically and biochemically isolated structures called dendritic spines. Long-term potentiation can be induced by specific stimulation paradigms resulting in the activation of specific biochemical signaling cascades. This has consequences on the spine structure, as well as on the trafficking and localization of neurotransmitter receptors, especially AMPA receptors. In this figure, we illustrate a number of events associated with synaptic plasticity. In the phenomenon of developmental synapse maturation many similar events are observed. Presynaptic changes: **(a)** Changes in high voltage-activated (HVA) voltage-dependent calcium channels (VDCCs) single-channel conductance affect Pr, changing the PPR and miniature postsynaptic current recordings (mPSCs) frequencies.** (b)** Changes in residual Ca^2+^ clearance affect Pr and therefore PPR, mPSCs frequencies. **(c)** Modulation of the vesicle fusion machinery would affect Pr and therefore PPR, mPSCs frequencies. **(d)** Depletion of the pool of readily releasable vesicles might reduce mPSC frequency and impact PPR. Due to synapses variability, could influence recorded mPSCs amplitude. **(e)** Changes in T-type low voltage-activated (LVA) VDCC properties would impact mEPSC frequency. Postsynaptic changes: **(f)** Nanodomains align active zones for vesicular release with postsynaptic receptors, constituting potential sites for the rapid exchange of receptors. **(g)** α-amino-3-hydroxy-5-methyl-4-isoxazolepropionic acid receptors (AMPAR) phosphorylation modulates single-channel conductance and **(g′)** membrane translocation. **(h)** Polyamine blockade: mediates rectification of Ca^2+^-permeable AMPARs, can produce changes in PPR. **(i)** Alteration of synapse geometry such as spine neck shortening can decrease resistance and facilitate cooperativity of synaptic responses.

Synaptic transmission between neurons consists of highly conserved electrical and chemical elements. Indeed, many of the key players in mammalian synaptic function and modulation were first identified through screens in invertebrates like fruit flies and nematodes (Gerschenfeld, 1973; Sweeney et al., 1995). Excitatory dendritic and somatic inputs result in the influx of positively-charged cations, which in turn leads to plasma membrane depolarization. At the axon hillock, this triggers the activation of voltage-dependent Na^+^ channels which propagates the ionic flux down the axon while simultaneously driving back-propagating voltage deflections in non-axonal compartments of the cell. Arriving at the presynaptic terminal, the membrane depolarization activates voltage-dependent Ca^2+^ channels, which allow Ca^2+^ to enter the terminal (Figure 1a). Increased intracellular Ca^2+^ mobilizes neurotransmitter-containing vesicles to fuse at active zone release sites on the presynaptic membrane, which are localized in direct apposition to postsynaptic specializations across the narrow synaptic cleft (~20 nm; Savtchenko and Rusakov, 2007; Südhof, 2012). Concentrations of transmitter in the synaptic cleft vary, but at a typical excitatory synapse, glutamate released from the presynaptic terminal quickly reaches concentrations >1 mM before dissipating with a decay time constant of ~1.2 ms (Clements et al., 1992). Neurotransmitter ligand-binding to postsynaptic receptor proteins evokes direct (ionotropic) ion flux or indirect (metabotropic) intracellular signaling cascades, which can lead to second-messenger signaling, structural growth, and protein synthesis. A number of human diseases are characterized by altered synaptic function, the development of therapeutic approaches and treatments for which depend on improving our understanding of the mechanisms that underlie synaptic transmission and its modulation.

### Development of Methodologies for Electrophysiological Investigation of Synaptic Events

The discovery by Katz and colleagues that spontaneous miniature end-plate potentials (MEPPs) recorded from the frog neuromuscular junction were similar in shape and amplitude to minimally evoked end-plate potentials in the presence of high Mg^2+^ or low Ca^2+^ led to the proposal that neurotransmission at synapses relies on the release of quantized packets of neurotransmitters in an all-or-none fashion (Del Castillo and Katz, 1954). This observation provided the basis for the quantal hypothesis, which posited that the measurement of a postsynaptic response (*I*) depends on the release probability (*Pr*) from a pool of releasable quanta (*N*) of defined quantal amplitude (*Q*). Therefore, the strength of an evoked postsynaptic response was modeled as:

I=QPrN

Although the precise definitions for these parameters may somewhat differ, this model provides a basis for understanding how changes in synaptic strength are measured. Indeed, these canonical electrophysiological approaches are still applied today and continue to provide insights into quantal parameters at the resolution of an individual synapse.

However, synapses in the central nervous system (CNS) are fundamentally distinct from peripheral neuromuscular junction synapses. The frog neuromuscular junction characteristically exhibits a low probability of release from defined inputs and high signal-to-noise ratio of quantal events that summate in a linear fashion (Mallart and Martin, 1967). In contrast, measurements of synaptic parameters in the CNS are confounded by a low signal-to-noise ratio that renders small-amplitude quantal events difficult to detect. Peripheral synaptic targets are innervated by individual or a relatively low number of inputs, whereas neurons in the CNS receive numerous synaptic connections, which impedes attribution of spontaneous quantal events to any single synapse (Megías et al., 2001; Gibbins and Morris, 2006). Further, in the CNS, excitatory and inhibitory inputs can terminate onto different neuronal compartments that, based on various geometric and resistive properties, modulate the amplitude of a measured response (Araya et al., 2006). Not only do different synaptic sites exhibit differences in quantal amplitude, but the probability of vesicular release can be non-uniform, with some presynaptic terminals disproportionately contributing to postsynaptic responses (Walmsley et al., 1988; Rosenmund et al., 1993). Together, these issues indicate that the foundational models based on the study of peripheral synapses are insufficient to fully interpret the function of central synapses.

### Techniques to Understand Synaptic Function in the Brain

Brain slice electrophysiology has been used experimentally for over 60 years, allowing for functional dissection of synaptic transmission at molecular, cellular, and network levels. First developed by Henry McIlwain in the late 1950s, these preparations allow for measurement of electrical potentials in maintained mammalian synaptic networks and have been an invaluable tool for virtually every field of neuroscience (Li and McIlwain, 1957; Yamamoto and McIlwain, 1966). Briefly, brain tissue is quickly extracted and cut into thick slices (250–400 μm). These slices are kept viable in oxygenated media containing appropriate ionic species, including Na^+^, K^+^, Mg^2+^, and Ca^2+^, mimicking the extracellular cerebrospinal fluid, termed artificial cerebrospinal fluid (ASCF). While various recipes have been used, ASCF generally contains high levels of Na^+^ and lower levels of other monovalent and divalent cations and anions. By replacing the endogenous constituents of the extracellular cerebrospinal fluid with known quantities, while using the internal ionic concentrations in the recording electrode to approximate transmembrane ionic gradients, the specific ion species that pass through synaptic receptor channels can be readily identified. Levels of extracellular Ca^2+^ are critical regulators of synaptic responses, and therefore altering Ca^2+^ concentrations can have a major impact on the release probability and degree of presynaptic vesicular mobilization, with higher concentrations resulting in increased presynaptic glutamate release and recorded postsynaptic current amplitude (Katz and Miledi, 1967; Malinow and Tsien, 1990; Llinás et al., 1992). Isolating currents and varying ionic concentrations to systematically shift the equilibrium potentials of specific ions allows extensive biophysical characterization of various channels and provides detailed insights into how chemical neurotransmission is converted to electrical impulses in the brain.

This basic approach has been modified in numerous ways to address questions that might otherwise be unfeasible in a traditional brain slice. For example, organotypic slice cultures, which result in an optically accessible monolayer that preserves much of the general organization and connection specificity of the brain, are particularly well suited for experiments in which repeated imaging is combined with recording, as well as for coculturing brain areas whose normal connectivity is severed by slicing (Gähwiler, 1981; Gähwiler et al., 1997). Some of these approaches can also be applied in intact or acutely isolated brain from amphibian and reptilian preparations that exhibit greater tolerance for hypoxia and temperature change (Wu et al., 1996; Bickler and Buck, 2007). Finally, with the advent to two-photon microscopy, the technique of “shadow-patching,” in which imaging and targeting of recording pipettes are performed through a cranial window, following the injection of fluorescent dye into the extracellular space to provide a three-dimensional inverted image of neuron locations, has improved the feasibility of whole-cell recording in the intact mammalian brain (Svoboda et al., 1997; Schmidt-Hieber and Häusser, 2013; Jayant et al., 2019). Such techniques preserve full network connectivity, but at the cost of reduced control over extracellular cerebrospinal fluid composition for drug application. Below, we discuss electrophysiology techniques used to record from brain tissue and address strengths and weaknesses associated with each method.

### Spontaneous and Miniature Synaptic Transmission: Interpretations and Implications for Network, Cellular, and Molecular Signaling

#### Spontaneous Post-synaptic Currents

Spontaneous postsynaptic currents (sPSCs) can be recorded while leaving network activity intact. Therefore, sPSCs provide information about the overall synaptic drive and activity of an intact neural circuit in the absence of exogenous artificial stimulation. Used in conjunction with miniature postsynaptic current recordings (mPSCs) in which AP generation is pharmacologically blocked, sPSCs allow for the dissection of overall network activity compared to stochastic spontaneous release and can inform whether alterations following an experimental condition result in changes in network properties or individual synaptic probabilities.

Recording sPSCs allows for measurement of the relative excitatory and inhibitory synaptic drive within a network, which have important functional implications for the overall output of a given neuron. Spontaneous current recordings can also be applied to probe the identity of the postsynaptic receptors when used in conjunction with pharmacological interrogation, or by varying the membrane potential of the recorded neuron to isolate the currents associated with a specific receptor. Ionotropic glutamate receptors pass cations, including Na^+^ and K^+^, with roughly equal permeability, producing a depolarizing current that reverses near 0 mV membrane potential. In contrast, activation of ionotropic inhibitory GABA_A_ receptors passes Cl^−^, and results in a current that reverses at the equilibrium potential for Cl^−^, usually near −65 mV. Consequently, by clamping the voltage of a cell at −65 mV, the driving force for the inhibitory component is effectively eliminated leaving only excitatory currents while depolarizing the cell to 0 mV can mute glutamatergic currents to reveal outward GABA responses. Comparison of the respective currents at each of these potentials allows for the calculation of the excitatory/inhibitory (E/I) ratio, which reflects the interplay of network activity that converges on an individual neuron. The balance of excitation vs. inhibition reflects the relative number of excitatory and inhibitory synapses and in healthy neurons, these levels of transmission are assumed to covary proportionally (Shu et al., 2003).

Changes in this balance can result in abnormal neural processing and computation. Synapse maturation during development leads to strengthening of cortical connectivity, reflected by increases in coherent neuronal network activity. In some neurodevelopmental disorders such as autism spectrum disorder (ASD), attention deficit hyperactivity disorder (ADHD), and schizophrenia, this balance is not maintained during synaptic pruning or the removal of aberrant synapses, for example, leading to a state of hyperexcitability that is characteristic of patients diagnosed with autism (Kehrer et al., 2008; Yizhar et al., 2011b; Nelson and Valakh, 2015). Consistent with an alteration in E/I balance, many neurodevelopmental disorders, including autism and schizophrenia, show co-morbidity with seizure-related pathologies, suggesting possible circuit dysregulation (Tuchman and Rapin, 2002; Friedman et al., 2008). However, a recent study systematically compared E/I balance in four different autism mouse models, and found that shifts in E/I balance, rather than being causative, may in fact reflect corrective homeostatic mechanisms designed to maintain stable overall synaptic drive to compensate for circuit dysfunction (Antoine et al., 2019).

Changes in the frequency of sPSCs are difficult to interpret since they may be affected by both pre- and postsynaptic mechanisms as well as alteration of overall network activity. Consequently, knowledge of the intrinsic and extrinsic network properties of the tissue preparation are important when considering the mechanisms that underlie changes in sPSCs. Further, in contrast to mPSCs, sPSCs show an increased probability of multivesicular release, which in turn can influence measured current amplitude. However, sPSC analysis is based on underlying assumptions of commonality between synchronous and spontaneous release mechanisms, presuming that these mechanisms exist in overlapping populations of synapses. Conversely, studies have demonstrated that ~40% of synapses in *Drosphila* exhibit dual-mode active zones, with ~36% of active zones showing preference for evoked release and 22% for spontaneous release alone (Melom et al., 2013). Indeed, highly-active synapses tend to show a preference for one mode of transmission over the other, indicating a specialization of synapses for different forms of synaptic communication (Peled et al., 2014). Consequently, localization of synaptic changes using sPSCs and mPSCs is problematic, due to differential expression of stochastic and synchronous evoked release mechanisms. Further, gross increases in levels of network activation can generate reverberatory activity, which can then mask alterations in synaptic frequency.

#### Miniature Post-synaptic Currents

Quantal release of neurotransmitter from the presynaptic terminal is stochastic when APs are eliminated using tetrodotoxin (TTX), a selective and potent blocker of voltage-gated Na^+^ currents. Under these conditions, spontaneous vesicular fusion events can be recorded from the postsynaptic neuron as mPSCs, resulting in either miniature excitatory post-synaptic currents (mEPSCs) or miniature inhibitory post-synaptic currents (mIPSCs). While AP-generated presynaptic responses strongly activate high-threshold P/Q- (Cav2.2) and N-type (Cav2.1) voltage-gated Ca^2+^ channels, stochastic Ca^2+^ influx for mPSC release is primarily mediated by low-threshold T-type Ca^2+^ channels including Cav3.1 and Cav3.2 (Catterall, 2011; Figure 1e). It should be noted, however, that mPSCs appear to consist of both Ca^2+^-dependent and independent events (Südhof, 2012). The frequency and amplitude of mPSCs have been used as proxies for the number of synapses and relative strength of synapses, respectively, made onto the postsynaptic cell (Segal, 2010). Stochastic spontaneous release suggests that a single mPSC event is likely the result of the release of a single vesicle. Consequently, recorded mPSC amplitude is a direct measure of ionic flux through postsynaptic receptors, with the underlying assumption that amount of neurotransmitter within a synaptic vesicle remains constant. However, variability between synapses that could also contribute to changes in average quantal amplitudes, as well as increases in either *Pr* or in the number of releasable quanta at a subset of synaptic inputs, will skew the distribution of mPSCs towards these inputs, meaning that changes in mPSC amplitude may not unequivocally reflect changes in postsynaptic receptors. Further, plasticity-induced changes in the geometry of a dendritic spine, such as changes that alter the resistance of a spine neck (Matsuzaki et al., 2001; Richards et al., 2005; Araya et al., 2014), may influence estimated measurements of *Q* independent of changes in postsynaptic receptor sensitivity (Figure 1i; Beaulieu-Laroche and Harnett, 2018; Cartailler et al., 2018).

Changes in the frequency of mPSCs have traditionally been associated with alterations in presynaptic function, arising from interactions between *Pr* and the pool of releasable quanta. To delineate the contribution of these presynaptic parameters, use-dependent pharmacological blockers may be employed to estimate the rate of spontaneous release that relies solely on *Pr* by measuring the decay of recorded currents (Atasoy et al., 2008; Sara et al., 2011). The recording of mPSCs in conjunction with the use of fluorescent molecule dyes (FM dyes) may also provide insights into changes in the synaptic vesicle pool (Gaffield and Betz, 2006). Therefore, a combination of techniques is required to ascribe a unitary underlying mechanism to a change in presynaptic function.

Postsynaptic mechanisms can also contribute to observed changes in mPSC frequency following pharmacological manipulation or induction of plasticity. N-methyl-D-aspartate receptors (NMDAR) and α-amino-3-hydroxy-5-methyl-4-isoxazolepropionic acid receptors (AMPAR) are two types of ionotropic glutamatergic neurotransmitter receptors. The conductance pore of the NMDAR can be blocked by extracellular Mg^2+^ ions when the cell sits at a hyperpolarized potential, rendering it functionally silent. The discovery that as excitatory synapses mature they can pass through a “silent synapse” stage in which they exclusively contain NMDARs with no AMPARs, provides a dramatic example of how an increase in the number and composition of postsynaptic receptors can result in an increase in mPSC frequency that is independent of *Pr* (Isaac et al., 1995; Liao et al., 1995). Indeed, insertion of postsynaptic AMPARs can increase the frequency of mPSC events by rendering the postsynaptic neuron more sensitive to presynaptic release at previously-silent synapses. While this phenomenon is quite well accepted in the developing nervous system, it has also been observed at hippocampal synapses in the adult brain (Sametsky et al., 2010; Glasgow et al., 2018).

Initial studies suggested that spontaneous events resulted from stochastic release of synaptic vesicles due to random Ca^2+^ fluctuations that engaged presynaptic mechanisms of vesicular release similar to evoked release (Fatt and Katz, 1950). Based on this assumption, miniature postsynaptic responses have been used as a proxy for evoked release mechanisms that define synaptic strength. However, more recent evidence suggests that spontaneous and evoked release may be distinct phenomena with different regulatory mechanisms and functional roles (Kaeser and Regehr, 2014; Kavalali, 2015). Evidence for this difference between spontaneous and evoked release has been demonstrated in multiple ways, including investigating vesicular release machinery (Schneggenburger and Rosenmund, 2015; Abrahamsson et al., 2017), the specific pool of vesicles released (Sara et al., 2005; Fredj and Burrone, 2009), postsynaptic targets (Atasoy et al., 2008; Sara et al., 2011), regulatory mechanisms (Nakamura et al., 2015; Maschi and Klyachko, 2017), and subcellular localization (Kneussel and Hausrat, 2016). Given such differences, it is highly likely that spontaneous and evoked release are mechanistically distinct, but the reasons for this apparent dissociation remain unclear.

Does spontaneous release contribute to ongoing activity? Spontaneous release of neurotransmitter is necessary for the maintenance of synaptic connections but is not required for initial synaptogenesis (Verhage et al., 2000; Varoqueaux et al., 2002). Consistent with a role in synaptic maintenance, CA1 hippocampal neurons require spontaneous postsynaptic receptor activation to maintain dendritic spines (McKinney et al., 1999; Segal, 2010). A number of excellent reviews have been published on the mechanisms underlying this form of synaptic plasticity (Turrigiano, 2008; Lisman, 2017; Diering and Huganir, 2018).

#### Synaptic Analysis Using Evoked Vesicular Release

Due to the independent mechanisms underlying spontaneous and evoked vesicular release, understanding the synaptic properties of a cell by measuring mEPSCs in TTX will not provide completely accurate insights into synaptic activation evoked by AP firing. Additionally, evoked stimulation typically excites multiple presynaptic axons, rendering postsynaptic recording as an integrated response to all vesicular exocytotic events. Minimal stimulation, in which the intensity of current delivered through a stimulating electrode is reduced to a level just above where complete failure of evoked release occurs, can be used to characterize individual synaptic properties in an identified pathway. However, the analytical strength of minimal stimulation experiments can be limited by many basic properties of fiber inputs, such as the heterogeneity of synapses formed by axons of different diameters, and the frequency of axonal conduction failures at branch points (Debanne, 2004; Kerchner and Nicoll, 2008).

Another powerful way to assess quantal synaptic characteristics in evoked stimulation studies is to induce asynchronous release, in which evoked synaptic release takes place over a much longer time period, allowing the discrimination of multiple individual quantal events (Otsu et al., 2004; Kaeser and Regehr, 2014). Substitution of Ca^2+^ with Sr^2+^ has been commonly used to transform synchronous release to asynchronous release, resulting in substantial reduction in the peak amplitude of evoked synaptic events while increasing the occurrence of asynchronous events that can be attributed to release from single synaptic sites (Goda and Stevens, 1994). Sr^2+^ enters the presynaptic terminal through voltage-dependent calcium channels (VDCCs) and activates the fast Ca^2+^ sensor, synaptotagmin-1, albeit with lower efficacy than Ca^2+^. Further, Sr^2+^ is extruded from presynaptic terminals less efficiently, resulting in a slowing of vesicular mobilization machinery (Xu-Friedman and Regehr, 2000). The quantal events induced by Sr^2+^-mediated asynchronous release can serve as a proxy for evoked EPSC strength since they appear to rely on the same release machinery and mechanisms as evoked release in the presence of Ca^2+^ (Kaeser and Regehr, 2014).

Use-dependent pharmacological blockers, such as MK-801 which stably occupies and occludes NMDAR channels in the open state, have also been applied to estimate the probability of release by measuring the decay of the curve as it relates to the reduced charge transfer of the evoked currents (Atasoy et al., 2008; Sara et al., 2011). However, typical pharmacological inhibition using bath application can result in changes in both presynaptic and postsynaptic neurons, confounding the spatial localization of function. To address the lack of spatial specificity of pharmacological blockers when used *in vitro* or *in vivo*, specific blockers can alternatively be included in the internal patch solution, circumventing network-wide alteration of function.

Quantal content at a given synapse is partially dependent on presynaptic factors, such as vesicular loading and exocytosis (Kaeser and Regehr, 2014). Estimations of multivesicular release can also confound the interpretation of single quantal events (Malagon et al., 2016). The rate of neurotransmitter reuptake by presynaptic terminals or astrocytes may also alter *Q* (Takamori, 2016). To isolate the postsynaptic contribution of *Q* it is possible to use optical uncaging, fast local perfusion, or microiontophoresis of pharmacological agents to control the concentration of neurotransmitter at a given synapse. Moreover, recent developments in fluorescent probes have facilitated estimation of quantal content using glutamate reporters such as igluSNfR (Marvin et al., 2013; Soares et al., 2017).

### Measuring Changes in Evoked Synaptic Strength

Changes in the relative strength of synapses are typically measured as alterations in postsynaptic current evoked in response to electrical, chemical, or optogenetic stimulation. Evoked stimulation using *in vitro* models provides robust and controlled levels of synaptic activation. Electrical stimulation has been used for several decades, however, it suffers from low cellular specificity, often requiring constant pharmacological antagonism of different receptor subtypes to record isolated excitatory or inhibitory synaptic currents. Further, stimulation of defined pathways results in simultaneous, synchronized release of presynaptic transmitter from multiple inputs, unlike physiologically-relevant conditions where synaptic release is far more desynchronized. Stimulation using novel chemogenetic and optogenetic actuators have helped to overcome some of the issues associated with low cell-type specificity characteristic of electrical stimulation but still presents important limitations. This section will address the various measures of evoked responses that have been used previously to spatially localize changes in function across a synapse and discuss some recent technical improvements for circuit dissection.

Synaptic plasticity involves the possibility of changes in both pre- and post-synaptic components. Coordinated changes in both compartments are often observed, which can make differentiating the specific roles of pre- vs. post-synaptic components in functional changes difficult to interpret accurately. While a few labs have been able to successfully record responses from axon terminals using patch clamp recordings (Alle and Geiger, 2006; Shu et al., 2006; Oltedal et al., 2007; Sasaki et al., 2012; Novak et al., 2013; Kawaguchi and Sakaba, 2015), the small size of presynaptic terminals (~1–2 μm) renders direct electrophysiological measurement difficult, and therefore the majority of research has relied on recordings from postsynaptic neurons to infer changes in presynaptic function. Such inference of presynaptic change relies on the protocols used to dissect out relative contribution of synaptic transmission. Below, we discuss the main techniques that have been used to identify the site and effectors of synaptic plasticity.

#### Paired-Pulse Ratio

APs travel down the axon to innervate the presynaptic terminal, resulting in the activation of voltage-dependent Ca^2+^ channels to trigger the mobilization and secretion of neurotransmitters. When two pulses are paired in quick succession (typically 20–100 ms), it is thought that residual Ca^2+^ left over from the first stimulus will transiently increase the release probability upon the second stimulus, termed short-term plasticity (Katz and Miledi, 1968; Zucker and Regehr, 2002). The relative peak amplitude of the first and second pulse, known as the paired-pulse ratio (PPR), therefore directly relates to presynaptic Pr. If the presynaptic terminal shows a high Pr, vesicular pools will be depleted following the first pulse, resulting in attenuated synaptic responses following the second stimuli and associated paired-pulse depression (PPD). In contrast, synapses showing low *Pr* can demonstrate facilitation following the second stimuli due to slow clearing of presynaptic intracellular Ca^2+^, a phenomenon termed paired-pulse facilitation (PPF). Consequently, changes in PPR have been interpreted to reflect presynaptic changes in *Pr*, although there are a variety of alternative mechanisms that may contribute to alterations in PPR (Figures 1b–d).

While PPR may be correlated with the relative *Pr* of a synapse, it is now clear that a diversity of other cellular mechanisms can influence the synaptic response to trains of input. The rapid reuptake of neurotransmitters from the synaptic cleft by transporters on astrocytes and synaptic terminals has been postulated to modulate PPF. Indeed, astrocytic coverage has been shown to reduce synaptic efficacy due to increased efficiency of glutamate clearance (Oliet et al., 2001). At the hippocampal Schaffer collateral synapse, the activation of glutamate uptake transporters by local CA1 astrocytes alters available glutamate levels (Bergles and Jahr, 1997), contributing to short-term changes in synaptic strength that include changes in PPR.

Rapid modification of postsynaptic receptors can also result in changes in PPR. Approximately 50% of all AMPA receptors are stably clustered within ~80 nm of the postsynaptic density at excitatory synapses, with the other fraction freely and quickly diffusing between them (Nair et al., 2013). Consistent with rapid exchange of AMPA receptors underlying short-term plasticity at individual synapses, experimentally crosslinking common AMPA receptor subtypes can modify forms of PPD, likely by blocking the swapping and postsynaptic removal of receptors (Heine et al., 2008). Rapid diffusion of AMPA receptors appears to increase the rate of recovery from PPD, which has been postulated to be due to endocytosis of desensitized receptors and replacement with naïve receptors (Constals et al., 2015). Additionally, it is possible that desensitized receptors may be exchanged for receptors with modified single-channel conductance (Figure 1g), which in turn can facilitate the postsynaptic response to pairs of presynaptic inputs that are differentially active across very brief intervals.

Synaptic strengthening has been linked to increased single-channel AMPA receptor conductance, resulting in enhanced ionic flow through an individual ionophore (Benke et al., 1998). Short term plasticity can elicit changes in the relative permeability of AMPA receptors to ionic flux. Calcium-permeable AMPA receptors (CP-AMPARs), which typically consist of GluA2-lacking AMPARs, are known to mediate fast excitatory synaptic transmission but are typically blocked by intracellular polyamines (PA; Burnashev et al., 1992; Anggono and Huganir, 2012). Endogenous cytoplasmic PA can tonically block CP-AMPARs to reduce the amplitude of evoked excitatory synaptic responses (Figure 1h). However, repetitive stimulation can relieve this PA block, resulting in a postsynaptic form of short-term plasticity (Toth et al., 2000). Neural activity can dynamically regulate PA synthesis to account for changes in PPR (Aizenman et al., 2003). Removal of the polyamine block through depolarization can result in attenuation of PPD and enhancement of PPF in cortical circuits (Rozov and Burnashev, 1999). Consistent with this, earlier work had demonstrated that PPR exhibits a strong voltage-dependence, and may rely on NMDA receptor activation (Clark et al., 1994). Further, PPR is decreased following postsynaptic activation of the Ca^2+^/calmodulin protein kinase 2 (CaMKII) signaling pathway in CA1 pyramidal neurons in response to Schaffer collateral stimulation, while simultaneously resulting in significant potentiation of synaptic responses (Wang and Kelly, 1996, 1997). CaMKII/calmodulin signaling can facilitate the function of CP-AMPARs, which in turn can mediate synaptic enhancement during protocols using PPR. Taken together, these findings suggest that changes in paired-pulse ratios can be expressed exclusively by the postsynaptic neuron through voltage-dependent removal of PA, independent of *Pr*.

#### AMPAR-to-NMDAR Ratio

Fast excitatory neurotransmission at central synapses is mediated by presynaptic release of glutamate, which in turn binds to specialized receptors on the postsynaptic neuron. Three major categories of glutamate-sensitive receptors, including AMPA, NMDA, and kainate receptors, have distinct kinetics that facilitate electrophysiological dissection of synaptic responses (Dingledine et al., 1999). AMPA receptors show large inward currents when recorded at relatively hyperpolarized membrane potentials, which are primarily mediated by Na^+^ influx with fast rise (2–7 ms) and decay kinetics (20–30 ms). In contrast, NMDA receptors, which are typically quiescent at resting potential and activate only upon depolarization-mediated removal of Mg^2+^ ionophore blockade, exhibit a slow rise (~20 ms) to maximal current, exhibiting a permeability for Ca^2+^, and a slow bi-exponential decay kinetic of 40–200 ms. Kainate receptors, which are active near rest potentials, show rapid rise-time accompanied by a slow decay constant that is sensitive to interactions with Neto1 auxiliary proteins (Straub et al., 2011); these have been reviewed in detail elsewhere (Huettner, 2003; Contractor et al., 2011). For the purpose of this review, we will focus on AMPA and NMDA receptor subtypes. Both receptor families are typically present at excitatory synapses in the CNS, and therefore the relative density of each receptor subtype is likely to play a key role in the function and plasticity of synaptic inputs.

Interrogation of postsynaptic receptor contribution to synaptic responses can co-opt voltage-dependence and decay kinetics to dissociate the relative contributions of receptor subtypes. Glutamate binds to both AMPA and NMDA receptors, with low and high affinity, respectively (Patneau and Mayer, 1990; Lester and Jahr, 1992). However, NMDARs do not flux ionic current when the membrane is near typical resting potential due to strong affinity for Mg^2+^ within the receptor ionophore, effectively blocking cationic movement upon glutamate receptor binding. In contrast, neuronal depolarization reveals an outward mixed synaptic current, consisting of both AMPAR-mediated and NMDAR-mediated components. Given the different time constants of NMDAR and AMPAR currents, the relative contribution of each receptor subtype can be readily dissected (Watt et al., 2000). While the initial component of the evoked EPSC shows a fast rise-time, including both NMDAR and AMPAR components, the rapid decay of AMPAR responses reveals a pure NMDAR-mediated current by >50 ms post-stimulus. This method of measuring AMPAR currents at hyperpolarized membrane potentials (typically −60 to −70 mV) and NMDAR currents around 50 ms post-stimulation at depolarized postsynaptic membrane potentials (typically +40 mV) allows for electrophysiological delineation of the glutamate receptor subtypes in the absence of pharmacological antagonists and is a fast and efficient measure of plastic changes in the composition of synaptic receptors.

#### Paired-Recordings of Synaptically-Coupled Neurons

The majority of studies focusing on synaptic transmission use bulk electrical stimulation of axonal fibers. Problematically, this can result in diffuse excitation of axons from multiple origins, confounding studies that routinely attribute stimulation to a single synaptic pathway. To overcome this issue, intracellular recording from pairs of monosynaptically-connected neurons provides an elegant technique to precisely measure circuit connectivity, presynaptic release mechanisms, and synaptic plasticity between defined neuron pairs. First developed in ganglionic recordings from *Aplysia*, paired recordings can effectively and robustly determine changes in both pre- and post-synaptic machinery at a small number of synaptic contacts, and allows for validation of presynaptic APs (Hughes and Tauc, 1968; Debanne et al., 2008). The benefits of this approach are 2-fold: APs of a single neuron can be measured as synaptic responses in a coupled neuron, and activity and relative timing of activation in two neurons can be correlated to facilitate investigation of activity-dependent forms of plasticity.

Coupled recordings can also be further validated using morphological reconstruction through inclusion of biocytin or another marker in the intracellular patch solution, allowing for elucidation of synaptic connectivity at both anatomical and physiological levels of investigation. Interestingly, dual patch recordings from coupled CA3 and CA1 neurons revealed that CA3 neurons form at a single CA1 contact *via* the Schaffer collateral, and show relatively low probability of transmitter release (Bolshakov and Siegelbaum, 1995). In contrast, pairs of excitatory cortical neurons are typically connected by 2–8 synaptic sites (Deuchars et al., 1994; Feldmeyer et al., 2005). Moreover, quadruple whole-cell recordings from layer V pyramidal neurons have demonstrated that clusters of bidirectional synaptic connections are more common than anticipated, and that neurons that share common presynaptic inputs are more likely to show correlated activity, providing a physiological basis for correlation-mediated activity-dependent synaptic plasticity (Song et al., 2005).

Study of presynaptic release mechanisms has been hugely impacted by the development of paired neuronal recordings. Transmitter release from the presynaptic axon terminal was traditionally believed to be mediated in an “all-or-none” manner due to initiation of APs, effectively functioning as a “binary” signal onto synaptically-connected postsynaptic neurons. However, through use of paired presynaptic axon terminal and postsynaptic somatic recordings, two groups were able to show that fluctuations in axon terminal membrane potential can potently enhance transmitter release, resulting in increased EPSC amplitude (Alle and Geiger, 2006; Shu et al., 2006). These findings suggest that presynaptic alteration of membrane potential can result in an “analog-like” modulation of synaptic transmission, and provide evidence that transmitter release from presynaptic terminals can be regulated at individual synapses through changes in both pre- and post-synaptic cellular excitability.

Are all synapses equally susceptible to plasticity? Excitatory synaptic responses elicited by electrical stimulation are typically used in studies of synaptic plasticity of defined pathways, yet activate multiple convergent inputs. Consequently, changes in synaptic strength cannot be attributable to any individual synapse. However, paired recordings of monosynaptically connected CA3 and CA1 neurons have demonstrated that a large subset of Schaffer collateral synapses fail to show potentiation following long-term potentiation (LTP) induction, highlighting a functional heterogeneity of LTP expression at different synaptic connections (Debanne et al., 1999). Therefore, observed changes in synaptic strength following LTP induction using electrical stimulation is likely to reflect large changes in a small number of individual synapses rather than a global facilitation of postsynaptic responses.

Paired recordings from synaptically-coupled neurons are a potent technique that can reveal a number of important mechanistic insights into synaptic transmission in acute brain slices. Indeed, it is a useful tool to study anatomical and physiological connectivity, presynaptic release function, and synaptic plasticity, and can be used to dissect relative contributions of individual synapses that would not be feasible using traditional bulk electrical stimulation.

#### Methods for Detecting Nascent or Silent Synapses

Changes in synaptic strength following the induction of LTP in the CA1 field of the hippocampus are primarily mediated by alteration of postsynaptic receptors (Figure 1g). While much work has focused on the recruitment of AMPARs to existing synapses, LTP may also reflect the addition of new AMPAR-containing synapses (Kerchner and Nicoll, 2008; Araki et al., 2015). However, the investigation of these previously-silent or nascent synapses is particularly difficult. Previous literature has demonstrated the existence of silent synapses or maturation of nascent synapses by exploiting a variety of methods, including coefficient of variation analysis, minimal stimulation, paired recordings, and glutamate photo-uncaging (Kerchner and Nicoll, 2008).

Excitatory glutamatergic synapses containing NMDARs but lacking AMPARs in acute adult hippocampal brain slices were initially described through analysis of trial-to-trial variability of EPSC amplitude (Kullmann, 1994). The overall variability of AMPAR-mediated and NMDAR-mediated EPSC responses can be expressed as a function of the coefficient of variability (1/CV^2^; the ratio of standard deviation of amplitude response to the mean amplitude of all events). Assuming that AMPARs and NMDARs are localized to all synapses, 1/CV^2^ should be equal for both receptor subtypes due to trial-by-trial variability of *Q* released from the presynaptic terminal. However, following LTP induction, 1/CV^2^ for AMPAR-mediated events is consistently decreased compared to NMDAR-mediated components (Kullmann, 1994). Synaptic responses exhibit a binomial probability distribution, which is a reflection of both the number of synapses and the *Pr* at each synaptic terminal. Consequently, in experiments when changes in synaptic strength following LTP induction were found to lead to a reduction in the overall variability of evoked AMPAR responses, this was initially erroneously attributed to increased *Pr*. Subsequent evidence revealed that the decreased variability was, in fact, attributable to AMPAR insertion into nascent synapses, effectively increasing the number (*N*) of synapses capable of responding (Lu et al., 2001). While silent synapses show no AMPA-mediated currents, they do contain NMDAR-mediated currents, measurable upon depolarization, that are unchanged following LTP induction (Kauer et al., 1988). Therefore, the NMDAR synaptic distribution does not appear to be altered by LTP induction, and is therefore useful as a stable electrophysiological measure for synapse function, even in the absence of AMPAR-mediated current.

Minimal stimulation to activate a few or even a single axonal fiber presumably elicits synaptic responses at a small number of postsynaptic sites. Following synaptic strengthening, the relative number of failures under minimal stimulation decreases despite using the same stimulus intensity, again suggesting that nascent synapses have been generated or that previously silent synapses have been unsilenced through insertion of AMPARs (Isaac et al., 1995). Similarly, intracellular recording from pairs of connected neurons has also been used to investigate synaptic unsilencing. Using paired recordings of CA3 neurons in organotypic hippocampal slices, Montgomery et al. (2001) showed that LTP can unsilence synapses (Montgomery et al., 2001). Paired whole-cell patch clamp recordings from two connected CA3 neurons resulted in NMDAR-mediated synaptic transmission at depolarized voltages, but no AMPAR-mediated synaptic responses at hyperpolarized potentials. Further, manipulations to increase the release probability of presynaptic terminals failed to elicit any postsynaptic response, suggesting a lack of AMPAR-containing postsynaptic sites. However, following a pairing protocol that facilitated NMDAR function coupled with presynaptic stimulation, AMPAR-mediated currents were readily observed with no detectable change in the NMDAR-mediated EPSC. Consistent with the unmasking of silent synapses, the failure rate of AMPAR-mediated EPSCs was significantly decreased by ~50% following LTP. These studies provide examples of methods to detect changes in silent synaptic connections and support the conclusion that activity can regulate synapse maturation through AMPAR insertion.

Isolated synaptic events can be simulated using photostimulation of caged compounds, such as MNI-glutamate, with diffraction-limited two-photon laser illumination. Caged compounds are biologically-active molecules that are rendered inert through a covalent attachment, which can be photolyzed with strong laser activation (Kaplan et al., 1978). A number of excellent reviews have been published on photo-uncaging in organotypic and acute hippocampal slices (Judkewitz et al., 2006; Reiner et al., 2015; Ellis-Davies, 2019). These findings show that changes in synaptic transmission can be due to a number of alterations in the postsynaptic neuron, including *de novo* spine formation and synaptogenesis.

#### Rapid Subunit Switching Without Receptor Exocytosis

Our focus thus far has compared pre-synaptic and post-synaptic mechanisms, however, recent studies have further parsed synaptic function, providing evidence that excitatory neurotransmission is mediated in the postsynaptic density at the nanometer scale (Eggermann et al., 2012). While previous models had primarily focused on the synapse as a whole, it is becoming exceedingly likely that excitatory synaptic transmission is organized as columnar nanodomains within the synapse. These “nanodomains” provide tight spatial constraints for postsynaptic activation, creating conditions in which presynaptic vesicular fusion occurs in extremely close proximity to the receptor site (Figure 1f; Choquet and Triller, 2013; Compans et al., 2016). What is the purpose of these nanodomains? While the answer remains elusive, it appears that certain types of neurotransmitter receptors, such as AMPARs, which exhibit relatively low affinity for glutamate, may be able to sit in reserve immediately adjacent to these nanodomains at the synaptic cleft, where they would contribute little or nothing to synaptic transmission. However, when mobilized during synaptic plasticity, they may be rapidly incorporated into the nanodomain. Consistent with this, various adhesion molecules that can mediate transsynaptic interactions and are well-known to have potent actions on the actin cytoskeleton, such as cadherin/β-catenin (Arikkath and Reichardt, 2008; Mills et al., 2017), neuroligin/neurexin (Chih et al., 2005; Haas et al., 2018), EphB/ephrin (Sheffler-Collins and Dalva, 2012), Slitrk/receptor protein tyrosine phosphatases (RPTPs; Yim et al., 2013), netrins (Goldman et al., 2013), integrins (Park and Goda, 2016), and others (Jang et al., 2017), have been found to be delivered to the synapse in response to activity and can affect rapid local structural reorganization (Benson et al., 2000).

Such local regulatory mechanisms are able to govern the density and spatial location of postsynaptic receptors at the synapse (Choquet, 2018). In contrast to previous models, which focused on a paradigm of receptor insertion at synapses, more recent work has begun to emphasize the importance of molecular-scale localization of excitatory glutamate receptors at postsynaptic slots, associated presynaptically with vesicular release sites and postsynaptically with intracellular scaffolding molecules like PSD-95. In turn, organization of the synapse can help to bring downstream signaling components into close proximity to excitatory ionic flux in the postsynaptic cell. Interestingly, other receptors such as NMDARs have higher affinity for their ligand and may therefore be less dependent on their sub-synaptic localization for signaling, allowing for extra-synaptic activation, potentially by different co-agonists (Rao and Craig, 1997; Dingledine et al., 1999; Papouin et al., 2012).

A nanodomain mechanism further raises the possibility of a role for the local trafficking of factors that can concomitantly regulate structural plasticity (Yamagata et al., 2003). Activity-dependent insertion or release of adhesion molecules such as protocadherins, cadherins, neuroligins, EphB, cerebellin, draxins, and others can promote specialization of postsynaptic and presynaptic densities (de Wit and Ghosh, 2016). These transsynaptic adhesion molecules span the synaptic cleft, and can rapidly modify the shape of synapses through intracellular interactions with the actin cytoskeleton (Murase et al., 2002; Okamura et al., 2004). Moreover, recent work has demonstrated that many adhesion molecules can interact with the N-terminal of glutamate receptors as well as synaptobrevin, suggesting that they may influence the local organization of nanodomains. Indeed, these findings indicate that synaptic structure is far more complex than originally proposed and that changes in synaptic strength may be mediated by rapid alteration of synaptic nanocolumns.

#### Extracellular Recordings

Extracellular field potential recordings *in vitro* offer access to identified neural circuitry for prolonged periods and facilitate pharmacological investigation, without dialyzing the intracellular contents of neurons. Measurements are typically made with glass electrodes filled with a highly-conductive solution such as 3M KCl, Na^+^, or ASCF and positioned in the dendritic field of neurons of interest to record alterations in local field potential, which results from the sum of electric current flow stemming from nearby sources. Local field potentials correspond to the concerted behavior, mainly synaptic, of multiple neurons and their processes proximal to the tip of the recording electrode.

This approach facilitates investigation of the “group average” as opposed to individual neurons, which may express various ionic channels, receptors, and other proteins differentially to neighboring neurons. As such, extracellular electrophysiological recordings can provide valuable insights to global changes in network properties following experimental manipulation. Moreover, because of their comparatively low level of invasiveness, field recording sessions can last for many hours, revealing so-called late-phase forms of LTP that persist for many hours and require protein synthesis (Nguyen et al., 1994).

*In vivo* field potential recordings permit repeated measurements of synaptic and network properties in the intact brain. Indeed, long-term recordings of neuronal activity have been maintained for up to several months. Recently, wireless electrophysiological recording systems have been developed and paired with video-based behavioral tracking for 24 h continuous observation over the course of 3 months (Grand et al., 2013), allowing for changes in neuronal excitability to be studied across long durations and under different behavioral contexts. Although these new techniques have tremendous potential, due to movement artifacts and other technical hurdles, it remains a significant technical challenge to perform robust long-term time-course recordings using *in vivo* recording electrodes.

### Optical Techniques for Investigating Synaptic Function

Recent advances in neuroscientific tools have allowed for the functional dissection of brain wiring with previously unparalleled specificity, temporal precision, and cell-type selectivity. In addition to an ever-growing number of cell-type-specific actuators and inhibitors, optical readouts have also greatly evolved in the past decade. The availability of optical recording techniques has presented the field with novel methods to record synaptic activity without the perturbations typical of more invasive techniques like intracellular patch clamp recording. Optical “read-outs” provide real-time information of cellular activity, and allow for precise, spatially constrained measurements of ongoing network function. Combining these technologies allows for new experimental approaches that can stimulate with light and measure functional changes optically. However, critical validation with electrophysiology is sorely lacking in many studies. The following section will briefly describe new readouts for cellular and synaptic activity, followed by descriptions of light-activated actuators and inhibitors of cellular function.

#### Calcium Indicators

Live imaging of intracellular Ca^2+^ dynamics owes much of its success to the efforts of Roger Tsien’s group starting in the early 1980s (Tsien, 1983; Grynkiewicz et al., 1985). A wide array of fluorometric and ratiometric dyes were developed with a range of affinities for Ca^2+^ binding that allowed for continuous monitoring of intracellular Ca^2+^ levels to investigate intrinsic and synaptic excitability in cultured cells and *in vivo*. Traditionally these dyes were loaded into neurons through a patch pipette, but a particularly powerful innovation involved the coupling of an acetoxymethyl (AM) ester to the dye, making it membrane permeant. AM-coupled dye could be injected directly into brain tissue to load hundreds of cells simultaneously. The AM ester would then be cleaved off the dye by intracellular esterases, trapping the activated fluorescent dye inside the cells (Garaschuk et al., 2006). However, toxicity and a lack of cell-type specificity associated with these dyes limited their applications in living tissue. Recent developments in both microscopy and GECIs, reviewed in detail elsewhere (Lin and Schnitzer, 2016), have allowed long-term analysis and investigation of synaptic strength. The most successful recent generation of GECIs, the GCaMP family, based on a circularly permutated green fluorescent protein fused with calmodulin and the M13 peptide from myosin light chain kinase, is now more sensitive than the original synthetic dyes, capable of detecting individual synaptic events and APs (Nakai et al., 2001; Tian et al., 2009; Dana et al., 2018). However, due to their slow kinetics of Ca^2+^ chelation, beneficial for relatively slow, laser-scanning microscopy approaches, many of these indicators offer relatively low temporal resolution of cellular excitation compared to more traditional electrophysiological methods. Multiple variants, with specifications for fluorescence change, spatial resolution, and response kinetics are constantly being developed. Recently, a set of four-color, spectrally-resolved Ca^2+^ indicators, XCaMPs that exhibit a large fluorescence signal change with more rapid kinetics has been reported (Inoue et al., 2019). Importantly, the spectral and kinetic properties of these new variants allow for better AP discrimination during trains and permit independent targeting of multiple genetically-defined cell types with different colors. The combination of whole-cell patch electrophysiology and new powerful GECIs variants with diverse characteristics substantially enables the subcellular localization of synaptic transmission and plasticity events within cells.

#### Optical Actuators

Optical stimulation employing light-activated actuators or inhibitors can elicit synaptic release or block synaptic input with precise spatial and temporal control. Early versions were based on neuronal ion channels modified to use light to gate conductances and depolarize neurons, but achievable time constants were slow and lacked specificity (Zemelman et al., 2003). The development of channelrhodopsin-2 (ChR2), a variant of an algal rhodopsin, shows millisecond precision and allows rapid, reversible control of neuronal or other cell type-specific activity. First described in 2005, ChR2 is a non-specific cationic channel that activates upon illumination with 473 nm light (Boyden et al., 2005). By combining viral delivery of ChR2 with Cre-LoxP mouse lines, optogenetic stimulation provides the ability to stimulate specific populations of genetically-defined neurons using light (Tsien et al., 1996; Yizhar et al., 2011a). This powerful technique has been used to dissect how different classes and ensembles of neurons regulate postsynaptic excitation, and more broadly how these neurons contribute to behavior (Glasgow et al., 2017). Further, recent work has used these optogenetic constructs delivered in retrograde viruses to facilitate pathway-specific excitation or inhibition (Schwarz et al., 2015), as well as ChR2-assisted circuit mapping (CRACM) of long-range projections (Petreanu et al., 2007).

Although extremely powerful, the use of optogenetics in measuring synaptic transmission, both *in vivo* and *in vitro*, faces most of the same issues that concern electrical stimulation, as well as additional concerns that include toxic light exposure and perturbation of normal cellular function. Variability in the level of ChR2 expression can reduce the utility of optogenetics as a tool to study synaptic transmission. Excitation of ChR2 in high-expressing neurons induces a large Ca^2+^ transient that travels throughout to the neuron to trigger APs with relatively short delays and high reliability, whereas low-expressing neurons routinely require extended blue light activation to elicit neuronal firing, that can result in phototoxicity and deleterious effects on cell health (Wade et al., 1988). Due to the relatively slow kinetics of ChR2, the resultant depolarization and firing is often delayed relative to the onset of light stimulation, obfuscating any link between the pulse of light stimulation and firing of the presynaptic neuron. Such findings suggest that high expression levels are required for studies investigating synaptic transmission; however, high levels of ChR2 expression have also been linked to neuronal defects and toxicity (Yizhar et al., 2011a; Miyashita et al., 2013). Further, compared to APs elicited by somatic current injection, light-evoked APs result in significantly higher levels of intracellular Ca^2+^, likely due to temporally-extended depolarization-mediated activation of voltage-gated Ca^2+^ channels (Zhang and Oertner, 2007). The reliance of presynaptic release machinery on the level of intracellular Ca^2+^ suggests that their saturation could alter *Pr* from the presynaptic terminal. These important characteristics of ChR2 require particular attention when using optogenetic tools to study synaptic transmission.

Optical stimulation can also impose artificial parameters on network activity. Due to light-mediated saturation of ChR2 currents in presynaptic inputs, a large number of presynaptic axons may be activated simultaneously by diffuse illumination. This mass excitation can impose non-physiological synchronous input on the postsynaptic neuron (Yizhar et al., 2011a). To mitigate the effects of simultaneous stimulation of presynaptic terminals, stable step-function opsins (SSFOs) can be used to generate a network-level depolarization or “up-state” (Berndt et al., 2009). Derived from the original ChR2, SSFOs exhibit temporally-extended decay kinetics (>20–30 min) and can be activated using a single pulse of blue light. This allows a single brief pulse (~5 ms) of blue light to depolarize neurons for extended periods of time and promote a network-level state of increased excitation. The decay kinetic of SSFOs can be enhanced through illumination with a brief pulse of red or orange light, effectively returning the neuron to its normal rest potential. Using these opsins with long decay kinetics offers a number of advantages for modulating network-level excitation, including promoting the generation of asynchronous APs for more physiological-like stimulation.

Conversely, neuronal silencing using inhibitory opsins has been used to reduce firing in a defined population of neurons. Effective optogenetic silencing is possible using the chloride pump red-light sensitive halorhodopsin derived from *Natronomas pharaonis* (NpHR; Zhang and Oertner, 2007). However, NpHR and its variants can alter some synaptic and cellular properties. Extended use of NpHR will shift the Cl^−^ homeostasis and reversal potential, as the neuron is unable to clear the anionic charge through Cl^−^ transporters. Consequently, at the offset of a light stimulus, neurons expressing NpHR will show a period of rebound excitation, releasing previously-silenced transmitter onto the postsynaptic neuron (Raimondo et al., 2012). In contrast, no rebound excitation was observed following inhibition with an alternate optogenetic inhibitor, archaerhodopsin from *Halorubrum sodomense* strain TP009, termed ArchT (Chow et al., 2010; Han et al., 2011). ArchT is a light-activated transporter that extrudes protons from the cytoplasm of neurons, which elevates pH when activated over long time-courses. Vesicular mobilization at presynaptic terminals is sensitive to changes in pH, suggesting that manipulating pH can alter vesicular dynamics and release of neurotransmitter.

In summary, these new neuroscientific tools to investigate synaptic transmission in the developing and mature nervous system will undoubtedly play critical roles in further understanding network connectivity and synaptic transmission; however, it is critical to understand the utility and limitations of any new tool. A carefully-planned combination of these optical tools alongside electrophysiological validation and calibration is best to ascertain how genetically-defined groups of neurons interact at the synaptic level.

## Discussion

Multiple electrophysiological techniques can be used to interrogate synaptic function in the developing and mature brain, and the emergence of new optical tools for both manipulation and measurement has allowed for unparalleled resolution of cellular processes underlying synaptic transmission. It is clear that understanding the contribution of pre- and postsynaptic mechanisms to synaptic plasticity must involve a number of diverse approaches to decipher how the brain changes individual synapses. Traditional interpretations of miniature EPSCs and paired-pulse protocols suggest that changes in these measures can reveal changes at presynaptic terminals. While alteration in AMPAR-to-NMDAR ratio and photo-uncaging have been understood to reflect postsynaptic changes, multiple studies in recent years have provided evidence that it is naïve to conclude that a phenomenon identified using a limited number of traditional approaches is purely pre- or postsynaptic.

Implementation of multiple electrophysiological methods, coupled with imaging techniques, is enormously beneficial for dissecting pre- and post-synaptic contributions to synaptic transmission and plasticity. As an illustrative example, we have recently demonstrated that the chemotropic guidance cue, netrin-1, is released from dendrites following NMDAR activation, and contributes to synaptic plasticity in the adult hippocampus (Glasgow et al., 2018). Netrin-1 increases mEPSC frequency with no accompanying change in mEPSC amplitude, which would traditionally be interpreted as an alteration in presynaptic function. However, we did not observe changes in PPR. In contrast, netrin-1 induced a significant increase in the AMPAR-to-NMDAR ratio, indicating a postsynaptic locus of action. Together, these findings suggested that netrin-1, which was been previously shown to direct cell and axon migration by regulating cytoskeletal reorganization in the developing nervous system, may promote the maturation of nascent synapses in the adult hippocampus, accounting for the increase in mEPSC frequency and the altered AMPAR-to-NMDAR ratio. Testing this idea, and consistent with a postsynaptic locus of action, failure rates using a minimal stimulation protocol were significantly decreased following the application of netrin-1, again supporting the conclusion that netrin-1 promotes the maturation and recruitment of nascent synapses. Through the combination of multiple different electrophysiological assays, the cellular and molecular mechanisms underlying netrin-1 mediated potentiation of synaptic transmission illustrate how the addition of new active synaptic connections can resemble alteration of presynaptic function, ultimately resulting in a facilitation of excitatory neurotransmission.

Recent technological developments have provided neuroscientists with an unprecedented tool-set to investigate synaptic transmission. The use of optical tools, in conjunction with classic electrophysiological methods, has provided new insights to traditional interpretation of synaptic function. By combining multiple approaches as described in this review article, convergent lines of evidence can be used to attribute changes in synaptic transmission to the pre- or postsynaptic compartment.

### Conclusions

Recent data confirms that synaptic transmission is an exceedingly complex phenomenon, subject to modifications in signaling at both pre- and post-synaptic sites. The development and refinement of whole-cell patch clamp electrophysiological techniques have greatly improved our understanding of how changes in the relative strength of synapses can contribute to various important functions mediated by the nervous system. Genetically encoded optical reporters and actuators have added powerful cell-type specificity to this analysis. However, interpretation of electrophysiological data requires careful attention to a number of parameters, including voltage-dependence, ionic flux, and experimental conditions. When possible, multiple experimental techniques should be employed to evaluate all possible loci of action. Together, convergent lines of evidence can reveal novel effector sites, and lead to re-evaluation of traditional interpretations and conclusions.

## Author Contributions

All authors contributed to the writing of this review article. The initial draft was written by SG, RM and JM in consultation with ER and TK. It was edited for accuracy and clarity by TK and ER. The summary figure was created by JM with feedback from the other authors.

## Conflict of Interest Statement

The authors declare that the research was conducted in the absence of any commercial or financial relationships that could be construed as a potential conflict of interest.
